# Are lifestyle changes from online information associated with discussing the information with a doctor? A cross -sectional study

**DOI:** 10.1371/journal.pone.0261471

**Published:** 2021-12-31

**Authors:** Tiki Celine Midthassel, Anne Helen Hansen

**Affiliations:** 1 Faculty of Health Sciences, Department of Community Medicine, UiT The Arctic University of Norway, Tromsø, Norway; 2 University Hospital of North Norway, Tromsø, Norway; Xiamen University - Malaysia Campus: Xiamen University - Malaysia, MALAYSIA

## Abstract

**Background:**

The prevalence of diabetes and the use of electronic health (eHealth) are increasing. Lifestyle changes in a positive direction may reduce morbidity and mortality in patients with diabetes. The main objective of this study was to test the association between lifestyle changes based on online information seeking and discussing the information with a doctor.

**Methods:**

In this cross-sectional study we used e-mail survey data from 1250 members of The Norwegian Diabetes Association, collected in 2018. Included in the analyses were 847 men and women aged 18 to 89 years diagnosed with diabetes and who reported use of eHealth within the previous year. We used descriptive statistics to estimate lifestyle changes based on information from the internet. Logistic regressions were used to estimate the associations between lifestyle changes after online information seeking and discussing the information with a doctor. Analyses were adjusted for gender, age, education, and self-rated health.

**Results:**

Lifestyle changes accomplished after online information seeking was reported by 46.9% (397/847) of the participants. The odds of changing lifestyle were more than doubled for those who had discussed information from the internet with a doctor (odds ratio 2.54, confidence interval 1.90–3.40). The odds of lifestyle changes were lower in the age groups 30–39 years and 60 years and over, compared to those aged 18–29 years (the reference group). Lifestyle changes were not associated with gender, education, or self-rated health.

**Conclusions:**

Our findings indicate that health-care professionals can play an important role in lifestyle changes additional to health-advice found on the internet. This study underlines the importance of easily accessible high-quality online information, as well as the importance of making room for discussing lifestyle in the clinical encounter.

## Introduction

### Prevalence of diabetes in Norway and globally

Diabetes is recognized as one of the fastest growing global health emergencies of the 21^st^ century. The global prevalence of diabetes was estimated to 463 million (9.3%) in 2019, and is expected to increase to 700 million (10.9%) in 2045 [1]. It is estimated that 244 000 people (4.7%) have diabetes in Norway; 28 000 type 1 diabetes (T1D), and 216 000 type 2 diabetes (T2D) [2]. Data from the Norwegian Prescription Database indicates that the prevalence of diabetes is increasing in Norway, as the number of people using anti-diabetic drugs has increased from 110 751 in 2004 to 207 517 in 2019 [3]. In recent years incident use of oral anti-diabetic drugs was stable or decreasing, which may indicate that the increase in diabetes incidence in Norway is levelling off [4]. People with diabetes have increased morbidity and mortality compared to the general population [5, 6]. Estimates from the Global Burden of Disease study 2015 shows that diabetes is the 10^th^ most important reason for disability adjusted life years (DALYs) in Norway [7]. Diabetes is a complex, chronic illness requiring continuous medical care with multifactorial risk-reduction strategies beyond glycemic control [8]. Most patients do not reach the combined treatment goals regarding HbA1c, systolic blood-pressure and LDL-cholesterol levels [9–12].

### Recommendations for lifestyle focus in people with diabetes

Lifestyle management is a fundamental aspect of diabetes care [13]. Physical activity, diet management and smoking cessation is recommended [13–15]. Exercise have shown several beneficial effects in people with diabetes included improved glycemic control (only T2D), beneficial effects on lipid-levels, increased insulin-response and a reduction in cardiovascular and total mortality [16–22]. Also, evidence is strong that medical nutrition therapy provided by registered dietitians is effective and essential [23]. Medical nutrition therapy is the process by which the nutrition prescription is tailored for each patient based on medical, lifestyle, and personal factors and is an integral component of diabetes management and diabetes self-management education [24]. Medical nutrition therapy improves glycemic control, and may also improve lipids, blood pressure, weight management, as well as decreasing the need for medication and reducing the risk of onset and progression of comorbidities [23]. Last but not least, smoking cessation is essential, as quitting smoking decreases the risk of both micro- and macrovascular complications of diabetes [25, 26]. Thus, there are strong indications that lifestyle changes in a positive direction may reduce morbidity and mortality in patients with diabetes.

### Increasing use of eHealth

The World Health Organization (WHO) defines eHealth as “the use of information and communication technologies for health” [27]. Health-related internet use has increased substantially in recent years [28–31]. 70–80% of internet users in Europe and the United States use the internet for health-related purposes [28, 30, 32–37]. Those more likely to seek health information online are women, younger people, people with higher education, higher household income, long-term-illness/heavy use of health-care services, and a subjective assessment of one’s own health as good [28, 30–38]. It was recently reported that 87% of Norwegians with T1D, and 78% with T2D use eHealth-services [39, 40].

In Norway, 98% of households have internet access [41] and 90% of the population between 16 and 79 years use the internet every day. Social media is used by 80% [42]. Among the Norwegian population over 15 years of age, 78% have reported using the internet for health-related purposes [30]. In year 2000, only 19% of the Norwegian population had searched for health-information online [30].

### Lifestyle changes after online information

Several studies have shown that internet-based interventions can promote health behavior change [43–45] regarding physical activity [46], diet, weight loss [47–49], and smoking cessation [50]. A quite consistent finding is that internet-based interventions that are interactive and tailored to individuals are generally more successful than those who are not, and that eHealth used with additional support, such as direct interaction with the health-care provider or in-person counseling, increase the effectiveness compared to stand-alone eHealth interventions [44, 45, 47–50]. There is no solid evidence whether this applies to people with T1D or T2D in Norway.

Among Norwegian internet-users the most used eHealth related activity was to read about exercise or diet, reported by 60% [30, 36]. A Norwegian study from 2007 found that 40% of those who had used internet for health-related purposes reported feeling inspired to change health behavior as a consequence [31]. Using internet- or mobile-based self-help programs to support health-behavior changes was reported by 17% of Norwegian internet users [36]. In the United States 43% of internet users reported using the internet to help with diet, weight and physical activity [51]. Using the internet for this purpose was associated with more fruit intake, more vegetable intake and more moderate exercise [51]. Since lifestyle focus is strongly recommended for people with diabetes, and the use of internet is widespread in Norway, we want to examine how use of the internet can affect lifestyle changes for the large group of people diagnosed with diabetes in Norway.

### Aim

The aim of this study was to investigate to what extent people with diabetes report lifestyle changes based on online information, and to examine associations between lifestyle changes and discussing the information with a doctor. Specifically, we aim to answer the following research questions: “To what extent do people with diabetes report lifestyle changes after online information?” and “How are lifestyle changes after online information seeking associated with discussing the internet information with a doctor?”

## Material and methods

### Data source

This cross-sectional study is based on the same data as the DIAcare project [40]. The project conducted a survey on use of eHealth among members of the Norwegian Diabetes Association (NDA) in January and February 2018. By December 31 2017, the organization had 33 908 members (53% women and 47% men). Around 30% of the members have T1D [52]. The Norwegian Centre for Research Data (NSD) assisted in the collection of data. Representatives from the NDA have been involved in the design, conduct, reporting, and dissemination of this research.

### Participants

Invitations to participate were sent by email to 5971 randomly selected members of the NDA (about 18% of all members). Information about the study was posted together with the email-invitation. It was not possible for the same member to fill in the questionnaire more than once. We sent one reminder to the non-respondents 15 days after the first request. A total of 1250 persons, aged 18–89 years answered the questionnaire, giving a minimum response rate of 21% (1250/5971). The real response rate is assumed to be higher as more than 400 servers were unable to deliver the invitation (bounce backs) [40].

**Fig 1:**
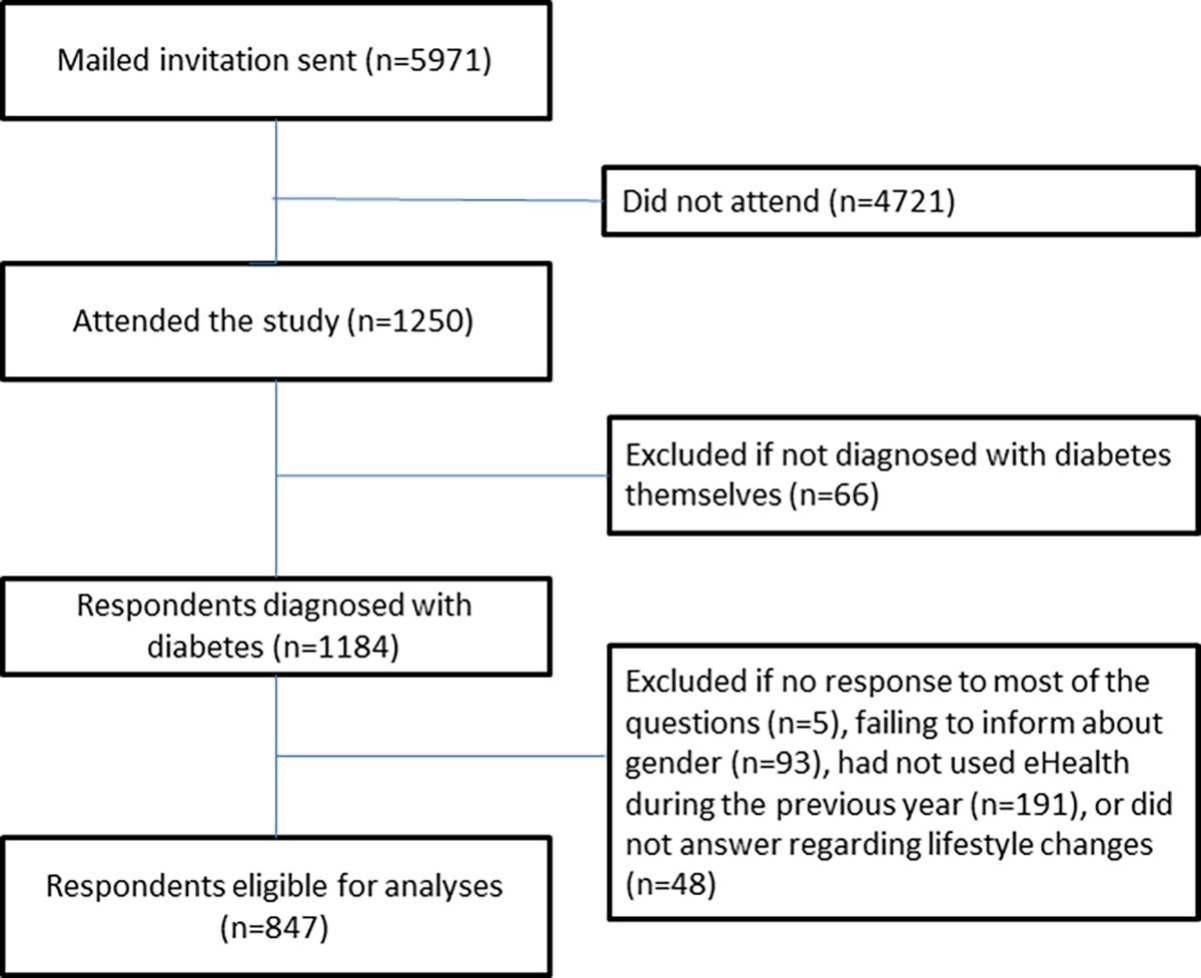
Flow chart of study population.

Starting from 1250, 66 participants were excluded for not having diabetes themselves (family members, health personnel, and others). Those who left out most of the questions (n = 5) and those who did not give information about gender (n = 93) were also excluded. Finally, we excluded those who had not used eHealth within the previous year (n = 191) and those who did not answer regarding lifestyle changes (n = 48). This left a sample of 847 respondents who were diagnosed with diabetes and had used eHealth during the previous year (Fig 1).

### Questionnaire and measures

Based on the specific objectives of our study we developed a tailored questionnaire, using questions from other published studies on health information seeking [53, 54]. The questionnaire (S1 Questionnaire) included questions about use of and experiences with eHealth and health care services, health status, and sociodemographic information. The questionnaire was tested and reviewed several times before data collection by persons diagnosed with diabetes and by experts from the DIAcare research group.

Regarding use of eHealth we included the two following questions; “Based on information you have found on the internet, have you changed your lifestyle?” and “Based on information you have found on the internet, have you discussed the information with a doctor?”. These variables were dichotomized by merging the four response options into “never” and “once, sometimes or often”, since our interest was if the respondents had ever made lifestyle changes or discussed online information with a doctor, rather than how often.

Age was grouped in 10-year intervals. The 4 education categories were labeled low (primary/part of secondary school), middle (high school), high (college/university less than 4 years), and highest (college/university 4 years or more). Self-rated health was measured with the item: “How do you rate your own health in general?” Response options were excellent, good, fair, bad, and very bad. Due to low numbers in the very bad category (4 respondents), we merged the bad and very bad categories.

### Analyses

Data were analyzed by descriptive statistics and logistic regressions. A multivariable regression model was constructed to analyze the associations between lifestyle changes based on information from the internet and the independent variables. The dependent variable was lifestyle changes based on online information seeking. The core independent variable was whether the participants had discussed eHealth information with a doctor. Adjustment independent variables were age, gender, education, and self-rated health. The independent variables were introduced collectively into the regression model.

As reference categories we used female gender, age 18–29 years, low education, excellent self-rated health and never having discussed information from the internet with a doctor. We used 95% confidence intervals and set P < .05 as significance level throughout the study. All analyses were performed using Stata version 15.1 (StataCorp LLC).

### Ethics

This study was approved by the data protection officer at the University Hospital of North-Norway (ref 2019/3761). The Regional Committee for Medical and Health Research Ethics (REK) found that an application for this project was not required according to the Norwegian Health Research Act (2015/1779/REK Nord). Informed consent was obtained from all participants. The data bureau NSD received no information about the participants other than the email addresses.

## Results

### Sample characteristics

Of the 847 participants included in this study, 438 were men (51.7%). Mean age of the participants was 53.9 years, 51.0 years for women and 56.6 years for men. Median age was 56 years. Over forty percent were aged 60 years and older (357/847, 42.2%). Half of the participants had T1D (428/847, 50.5%), 47.7% had T2D (404/847), and 2.8% had other types of diabetes (24/847). Four of the participants reported having both T1D and T2D, and two reported having both gestational diabetes and T2D. Therefore, six of the participants entered into more than one group regarding type of diabetes (Table 1).

**Table 1:** Sample characteristics.

	n (%)
**Have you discussed information from the internet with a doctor? (n = 845)**	
Never	443 (52.4)
Once/sometimes/often	402 (47.6)
**Have you changed your lifestyle based on information from the internet? (n = 847)**	
Never	450 (53.1)
Once/sometimes/often	(46.9)
**Gender (n = 847)**	
Female	409 (48.3)
Male	438 (51.7)
**Age in years (n = 847)**	
18–29	73 (8.6)
30–39	87 (10.3)
40–49	127 (15.0)
50–59	203 (24.0)
60–69	229 (27.0)
≥ 70	128 (15.1)
**Type diabetes (n = 847)**	
Type 1	428 (50.5)
Type 2	404 (47.7)
Other (including gestational diabetes)	24 (2.8)
**Education (n = 826)**	
Low	73 (8.8)
Middle	240 (29.1)
High	262 (31.7)
Highest	251 (30.4)
**Self-rated health (n = 841)**	
Excellent	118 (14.0)
Good	424 (50.4)
Fair	212 (25.2)
Poor/very poor	87 (10.3)

Most of the participants, 62,1% had education from college or university (high or highest education group, 262/826, 31.7% and 251/826, 30.4%, respectively). Half of the participants rated their own health as good (424/841, 50.4%). Just over half of the participants had never discussed information from the internet with a doctor (443/845, 52.4%). Lifestyle changes accomplished after online information seeking was reported by 46.9% (397/847).

### Lifestyle changes after online information

The odds of changing lifestyle after online information was more than doubled for people who had discussed information from the internet with a doctor, compared to those who had not (OR 2.54, CI 1.90–3.40).

People aged 60–69 years and 70 years and over had significantly lower odds of reporting lifestyle changes after online information, compared to people aged 18–29 years (odds ratio (OR) 0.54, CI 0.31–0.95 and OR 0.42, CI 0.23–0.80, respectively). Persons aged 30–39 years also had significantly lower odds of lifestyle changes compared to persons aged 18–29 years (OR 0.49, CI 0.25–0.97). The age groups 40–49 years and 50–59 years did not have significantly lower odds of lifestyle changes compared to the reference group. Gender, education, and self-rated health were not associated with lifestyle changes (Table 2).

**Table 2:** Odds of changing lifestyle after online information.

	OR^a^	P value	95% CI^b^
**Have you discussed information from the internet with a doctor?**			
Never	1.0		
Once/sometimes/often	***2*.*54***	***<0*.*001***	***1*.*90–3*.*40***
**Gender**			
Female	1.00	^c^	
Male	0.99	0.95	0.74–1.33
**Age in years**			
18–29	1.00		
***30–39***	***0*.*49***	***0*.*04***	***0*.*25–0*.*97***
40–49	0.79	0.46	0.43–1.46
50–59	0.74	0.30	0.42–1.30
***60–69***	***0*.*54***	***0*.*03***	***0*.*31–0*.*95***
***≥ 70***	***0*.*42***	***0*.*01***	***0*.*23–0*.*80***
**Education**			
Low	1.0		
Middle	1.04	0.89	0.60–1.81
High	0.95	0.84	0.54–1.64
Highest	1.01	0.97	0.58–1.77
**Self-rated health**			
Excellent	1.0		
Good	1.12	0.60	0.73–1.74
Fair	1.07	0.77	0.67–1.73
Poor/very poor	1.35	0.32	0.75–2.43

^a^OR: odds ratio.

^b^CI: confidence interval.

^c^Not applicable (reference group).

Statistically significant findings are written in bold and cursive.

## Discussion

### Principal findings

Almost half of the participants (397/847, 46.9%) reported that they had made lifestyle changes based on online information, once or more. The odds of making lifestyle changes were higher when discussing information from the internet with a doctor, and lower for age groups ≥ 60 years. Those aged 30–39 years had significantly lower odds of lifestyle changes after online information compared to persons aged 18–29 years. Lifestyle changes from online information were not associated with gender, education, or self-rated health.

### Strengths and limitations

One of the study strengths is the recruitment of a quite homogenous group of study participants from a patient organization, covering all Norway and a wide age span. Also, the focus on a scarcely investigated research area is a strength. Another strength is the use of a systematically tested comprehensive questionnaire. The cross-sectional study design based on self-reported data gave us the opportunity to investigate lifestyle changes after online information on a general basis, rather than focusing on one type of lifestyle change after the use of one specific webpage/app/internet-intervention.

The low participation rate is the main limitation of this study. This was problematized by Hansen et al. in the DIAcare project [40]. They therefore compared respondents who did not respond initially but eventually consented with early respondents, assuming that late respondents were more similar to non-respondents, finding that older people (60 years and over) dominated among the late respondents. Younger people may thus be overrepresented in this study. However, participation in studies are generally lower in younger people [55], which may contribute to level out possible overrepresentation of younger people. Nevertheless, generalization should be made with caution.

The percentage of people with T1D is higher in our study than in the general diabetes population in Norway (Table 1) [2]. However, this study does not claim to be representative for T1D or T2D in particular. Rather, the overall general perspective of lifestyle changes obtained from online information approaches T1D as well as T2D as chronic disease groups in need of long term follow up and lifestyle considerations.

In questionnaire data there is always a potential for recall bias, which can lead to under- or overreporting. For this study, it would be very difficult to obtain objective measures instead of self-reported ones, as it would interfere with participants’ privacy as well as be very time- and cost consuming. There is also a risk that such measures would not necessarily provide objective measures in this research area, and studies have suggested that self-reported data are valid when it comes to Web-based eHealth solutions [56]. Our validation and testing of the questionnaire minimize the risk of misunderstandings and different interpretations by the respondents [40].

The wide and general perspective of this study, as we have so far considered a strength, might also be considered a limitation. We do not have specific information about the nature of the reported lifestyle changes, nor about the content of the patient-doctor discussion. Consequently, we do not know which specific lifestyle topics related to online information that were discussed in the clinical encounter. This study of lifestyle changes based on online information as a phenomenon should be followed up by studies on specific lifestyle changes for people diagnosed with diabetes.

A limitation of a cross-sectional study design is that it precludes any causal interpretation.

### Lifestyle changes from online information

Nearly half of the participants in this study reported that they once or more had made lifestyle changes based on online information. A Norwegian study with data from 2007 found that 40% of those who had used internet for health-related purposes reported feeling inspired to change health behavior as a consequence [31]. A US study found that in 2011, 43% of internet users used the internet to help with diet, weight and physical activity and that using the internet for this purpose was associated with more fruit- and vegetable-intake and more physical activity [51]. These numbers are just slightly lower than what we found. However, none of these studies reported on how many who had actually changed their lifestyle based on online information. In contrast to these studies, our study is conducted among people with diabetes, and previous research has indicated that persons with chronic diseases tend to use the internet for health purposes to a higher extent than the general population [30, 32, 37]. This might be the main explanation why the proportion of people reporting lifestyle changes in this study is higher than in the general population. Although none of the studies mentioned above are directly comparable to ours, they support the finding that internet can play an important role in lifestyle changes.

### Lifestyle changes and discussing online information with a doctor

The odds of making lifestyle changes after online information seeking more than doubled for people who had discussed information from the internet with a doctor. Metanalyses have indicated that face-to-face interactions can increase the effectiveness of internet interventions [45, 49]. A recent qualitative study among obese diabetes patients who had participated in an eHealth-intervention for weight loss, found that the most important driver in long-term weight loss was a strong relationship with a healthcare professional [57]. Our findings are in line with this research, emphasizing that health-care professionals can play an important role in lifestyle changes additional to health-advice found on the internet. In general, there are evidence that eHealth services are additional rather than alternative to support provided by traditional health care services [40]. Our findings imply that it is important that health personnel do discuss lifestyle advice with patients.

### Lifestyle changes and age

The odds of making lifestyle changes based on online information was lower in people aged 60 years and over. Previous research indicate that younger people use the internet for health purposes more than older people [28–32, 34, 35, 51]. There are also indications that younger people to a larger extent trust information from the internet, as around 80% of young French adults (mean age 22.6 years) trusted health information from the internet [58], whereas around 40% of older people in the Netherlands (mean age 72.0 years) trusted this information source [53]. More use of eHealth and greater trust in internet information among younger people might make them more able to make lifestyle changes based on internet information.

A quite surprising finding in our study was that people aged 30–39 years reported significantly less lifestyle changes compared to people aged 18–29 years. For the use of eHealth in general we have not found any reports that people aged 30–39 years are less active. Rather, Andreassen et al. reported in 2007 that the 30–44 age group included the most active eHealth users in seven European countries [32]. A possible explanation of our finding could be that people on their 30s are strongly occupied with career and family, rather than focusing on lifestyle changes.

### Gender, education and self-rated health

We found no associations between lifestyle changes and gender, education or self-rated health. Women tend to use the internet for health purposes more than men [29, 30, 32–37]. However, using the internet for health purposes does not necessarily lead to lifestyle changes. A Danish study among obese diabetes patients, found no significant gender differences in average weight loss after an eHealth intervention [59]. This finding is in line with ours and may indicate that men and women are equally able to make lifestyle changes from internet information. As both studies are conducted among people with diabetes in Scandinavia, the findings are not necessarily representative for other populations.

Prior research quite consistently indicates that people with higher education use the internet for health purposes to a greater extent [28, 31–34, 37], including using the internet to support exercise or diet [36, 51]. A possible explanation to why we did not find differences according to education could be related to adaption time for the use of internet for health purposes. People with higher education are often early adapters to novel technology. Now, when consulting the internet is more common and widespread [28–31], the differences may even out. Therefore, differences according to education might be larger in earlier studies, compared to more recent ones. Our results might also be influenced by the high amount of participants with college or university education (62.1%), compared to the general Norwegian population 34.6% [60]. Also, the percentage of persons with T1D is higher in our study than in the general diabetes population in Norway [2], and there are indications that that people with T1D are higher educated than people with T2D [39]. This may in turn partly be explained by socio-economic inequalities in the prevalence of T1D and T2D, as well as by the higher age in the T2D group due to a higher average age by the time of diagnosis. In Norway and many other countries older people have a lower level of education compared to younger residents. These factors make our findings regarding education inconclusive.

We found no associations between lifestyle changes and self-rated health. This is in line with Alvarez-Galvez et al., who found no significant relationship between self-rated health and the use of internet for health purposes [37]. We were not able to find solid studies investigating the associations between self-rated health and lifestyle changes based on internet information among people with diabetes.

### Further research

To extend our study, future research could investigate what kind of lifestyle changes people make from internet information, as well as the amount of changes and whether they sustain. It would also be interesting to investigate the relation between any particular information obtained from the internet, and the discussion in the clinical encounter. Likewise, investigations of possible facilitators and barriers to discussing information from the internet with health-care providers are scarce. Further research should also seek to find possible explanations to our finding that people aged 30–39 years report less lifestyle changes.

## Conclusions

Our findings confirm that internet can play an important role in lifestyle changes among people with diabetes, and that discussing internet information with a doctor more than doubles the odds of lifestyle changes. It is thus important that high-quality updated online information is easily available for patients. This study also indicates that health-care professionals can play an important role in lifestyle changes additional to health-advice found on the internet, underlining the importance of making room for discussing lifestyle advice in the clinical encounter.

## Supporting information

S1 Questionnaire (PDF)
